# A Retrospective Observational Study of the Microbial Etiology and Antimicrobial Susceptibility Patterns of Blood Cultures From ICU Patients at a Healthcare Facility in North India

**DOI:** 10.7759/cureus.57356

**Published:** 2024-03-31

**Authors:** Amit Kumar, Nikhil Raj, Sangeeta Singh, Anupam Das, Vikramjeet Singh, Manodeep Sen, Jyotsna Agarwal

**Affiliations:** 1 Microbiology, Dr. Ram Manohar Lohia Institute of Medical Sciences, Lucknow, IND

**Keywords:** mbl, esbl, bacteriological profile, antibiogram, blood culture profile, bloodstream infection

## Abstract

Introduction

Bloodstream infections (BSI) are a leading source of fatalities and morbidity in hospitals. However, the clinical spectrum and antimicrobial resistance differ globally. Identifying the pathogenic spectrum and variations in antibiotic resistance is crucial for controlling BSI and preventing inappropriate antibiotic use.

Material and methods

This retrospective observational study was conducted at the Dr. Ram Manohar Lohia Institute of Medical Sciences, Lucknow, UP, India, for one year between June 2022 and June 2023. A total of 669 adult patients' blood cultures were obtained from ICUs. Blood culture was done using a BacT/Alert 3D (BioMérieux SA, Marcy-l'Étoile, France) automated system. Identification of the bacterial as well as fungal isolates was done using matrix-assisted laser desorption ionization-time-of-flight mass spectrometry (MALDI-TOF MS), and the antimicrobial susceptibility profile was analyzed using the VITEK 2 Compact system (BioMérieux SA).

Results

Of the 669 blood culture samples, 213 (31.8%) showed bacterial or fungal growth. Of these 213 isolates, the most common isolate was coagulase-negative *Staphylococci *(21.6%), followed by *Klebsiella pneumoniae *(19.3%)* *and Acinetobacter* spp. *(17.8%). The majority of gram-negative bacteria were resistant to most drugs, and vancomycin and linezolid were both effective against the majority of gram-positive bacteria.

Conclusion

The current study found that septicemia was more frequently caused by gram-negative bacteria than by gram-positive bacteria. Blood cultures are always necessary in cases of suspected septicemia, and once the antimicrobial susceptibility profile of the pathogen causing septicemia has been determined, suitable antimicrobials should be prescribed and used to lower the antimicrobial resistance burden.

## Introduction

Bloodstream infection (BSI) is a significant cause of illness and death worldwide [1]. It can be primary or secondary depending upon the origin, primary if unidentified, and secondary when the source is documented [2]. The term 'bacteremia' describes the growth and presence of bacteria in the blood, whether sepsis symptoms are present or not. *Enterococcus*, coagulase-negative *Staphylococci *(CoNS), and *Staphylococcus* are the gram-positive bacteria that are most frequently isolated. Meanwhile, *Escherichia coli*, *Klebsiella pneumoniae*, *Pseudomonas aeruginosa*, and *Acinetobacter baumannii* are commonly found among gram-negative bacteria [3]. Nosocomial infections have been found to affect up to 20% of patients in ICUs [4]. Within the first month of admission to the ICU, about 7% of incidents result in BSI. When central venous catheters and other invasive devices are used in patients with critical illnesses, the risk of infection increases [5]. Acquiring a BSI leads to longer ICU stays and higher healthcare costs [6]. The International Surviving Sepsis Campaign recommends the immediate initiation of empirical broad-spectrum antibiotics to target all potential pathogens [7]. To reduce antimicrobial resistance, broad-spectrum antibiotics should be used carefully. In intensive care settings, the usage of broad-spectrum antimicrobials is usually high, which increases the risk of antibiotic-resistant bacterial infections [8]. A blood culture has a very high positive predictive value for diagnosing BSI. Prompt diagnosis and appropriate antibiotic therapy can reduce the burden of BSI in the ICU [8]. To evaluate the burden of BSI, this study was carried out to observe the pathogens causing BSI and their antimicrobial resistance.

## Materials and methods

This retrospective study was carried out in a hospital setting at the Dr. Ram Manohar Lohia Institute of Medical Science, located in Lucknow, UP, India. The data were collected by reviewing the medical records of 669 patients hospitalized in the ICU between June 2022 and June 2023. All blood culture samples taken from hospitalized patients in different ICUs were included. Cultures with mixed bacterial growths, repeat samples from the same patient, and those samples that produced contaminants (diphtheroids, *Micrococcu*s, and aerobic spore *Bacillus*) were not included in the study. Blood was collected using aseptic techniques with blood culture bottles, and the bottles were incubated in BacT/ALERT 3D (BioMérieux SA, Marcy-l'Étoile, France), a system for automatically detecting microbial growth. When the bottle flagged positive, it was subcultured on blood agar (HiMedia Laboratories Pvt. Ltd., Mumbai, MH, India) and MacConkey agar (HiMedia Laboratories Pvt. Ltd.), and plates were incubated overnight at 37°C. Gram staining was done from the colony, and after the identification of bacterial or fungal organisms from Gram stain, species identification was done using matrix-assisted laser desorption ionization-time of flight mass spectrometry (MALDI-TOF MS), which is a rapid method for automated bacterial identification based on protein profiling of bacteria. The detection of the antimicrobial susceptibility of isolates was done using the VITEK 2 Compact system (BioMérieux SA), an automated, advanced expert system. For testing susceptibility, the Clinical Laboratory Standards Institute (CLSI) 2022 recommendations were followed [9].

The antibiotics that were tested for bacterial isolates included cefoxitin, erythromycin, clindamycin, tetracycline, ciprofloxacin, chloramphenicol, vancomycin, linezolid, gentamicin, teicoplanin, ampicillin-sulbactam, ceftazidime, imipenem, piperacillin-tazobactam, and amikacin. As per the CLSI M44 recommendation, antifungal susceptibilities to amphotericin B (AMP B), fluconazole caspofungin, flucytosine, micafungin, and voriconazole were conducted on all *Candida* species using the VITEK 2 Compact system [10]. *Escherichia coli* (ATCC-25922), *Staphylococcus aureus *(ATCC-25923), *P. aeruginosa* (ATCC-27853), and *Candida albicans* (ATCC-10231) were used to evaluate the quality control of VITEK 2 Compact antimicrobial susceptibility testing (AST) cards for each new lot. The existence of organisms that produce metallo β-lactamase (MBL), extended-spectrum β-lactamase (ESBL), and methicillin-resistant S. aureus (MRSA) was confirmed by the VITEK 2 Compact system. The statistical software SPSS Statistics version 26 (IBM Corp., Armonk, NY, USA) was used to analyze and interpret the data produced in this investigation. For descriptive purposes, frequencies for categorical variables were calculated. Data on antimicrobial susceptibility were presented as a percentage.

## Results

A total of 669 samples from probable septicemia cases admitted to different ICUs were received during the study period. Out of them, 213 (31.8%) had microbiological growth detected. Among the positive-flagged samples, 66.2% were male and 33.8% were female. All positive-flagged samples were monomicrobial. Out of all positive cultures, 112 (52.6%) were caused by gram-negative bacteria, 75 (35.2%) by gram-positive bacteria, and 26 (12.2%) by fungal isolates. *Klebsiella pneumoniae* (41, 19.3%) was the most common isolate among gram-negative bacteria, followed by *Acinetobacter* species (38, 17.8%) and *P. aeruginosa* (19, 8.9%), which appeared as the next common pathogens. The gram-positive cocci that were most common were CoNS (46, 21.6%), followed by *S. aureus* (20, 9.4%). *Candida tropicalis* (10, 4.7%) was the most prevalent organism among the fungal isolates, followed by *Candida auris* (seven, 3.3%) (Table 1).

**Table 1:** Distribution of the isolated organism Total no. of isolates: n = 213 (100%) CoNS: Coagulase-negative *Staphylococci*

Organism	No. of isolates n (%)
Gram-positive organism	
CoNS	46 (21.6)
Staphylococcus aureus	20 ((9.4)
*Enterococcus* species	9 (4.2)
Gram-negative organism	
Klebsiella pneumoniae	41 (19.3)
*Acinetobacter* species	38 (17.8)
Pseudomonas aeruginosa	19 (8.9)
Escherichia coli	5 (2.3)
Citrobacter freundii	3 (1.4)
Providencia stuartii	3 (1.4)
Elizabethkingia anophelis	1 (0.5)
Serratia marcescens	1 (0.5)
Stenotrophomonas maltophila	1 (0.5)
Fungal Spp.	
Candida tropicalis	10 (4.7)
Candida auris	7 (3.3)
Candida albicans	3 (1.4)
Candida parapsilosis	3 (1.4)
Candida kruesi	2 (0.9)
Candida lusitaniae	1 (0.5)

**Figure 1:**
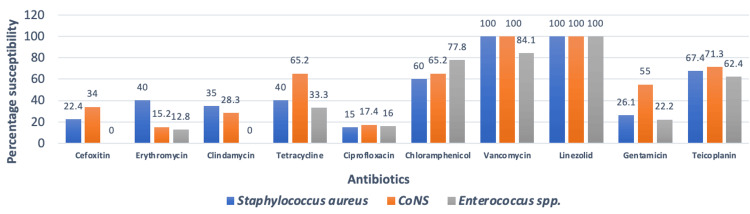
Antibiotic sensitivity pattern of gram-positive organisms CoNS: Coagulase-negative *Staphylococci*

In the case of gram-positive isolates, linezolid, and vancomycin were 100% susceptible to CoNS and *S. aureus*. Around 77.6% of *S. aureus *and 66% of CoNS were methicillin-resistant. For *S. aureus*, ciprofloxacin sensitivity was the lowest. The least sensitive drug for CoNS and *Enterococcus* species was erythromycin. *Enterococcus* species was 100% sensitive to linezolid. Sensitivity to vancomycin was only 84.1% among the *Enterococcus* species (Figure 1).

**Figure 2:**
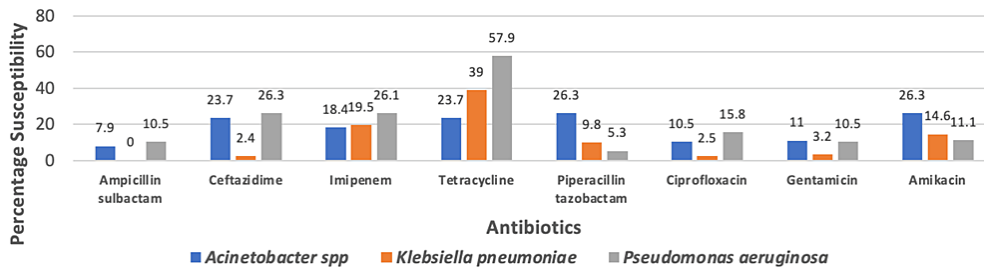
Antibiotic sensitivity pattern of gram-negative organisms

Our study reveals that the majority of gram-negative bacteria display pan-drug resistance. The most sensitive antibiotics for the *Acinetobacter* species were amikacin and piperacillin-tazobactam. Tetracycline was the antibiotic that was most sensitive to *P. aeruginosa* and *K. pneumoniae*. In our study, ceftazidime (2.4%) was the least sensitive antibiotic when used against *K. pneumoniae* (Figure 2).

Most of the gram-negative organisms were producers of MBL, with a percentage of 64.3%. Metallo-β-lactamase was found in 71% of *Acinetobacter spp*. and 65.9% of *K. pneumoniae*. About 19.6% of gram-negative bacteria were ESBL producers. Extended-spectrum β-lactamase was found in 26.8% of *K. pneumoniae* cases. Out of all the gram-negative isolates tested, 16.1% were found to neither produce MBL nor ESBL (Table 2).

**Table 2:** Distribution of MBL and ESBL producers among gram-negative organisms MBL: Metallo-β-lactamase, ESBL: Extended-spectrum β-lactamase

Organism (n)	MBL producer n (%)	ESBL producer n (%)	Non-MBL and non-ESBL producer n (%)
*Acinetobacter* species (38)	27 (71.0)	5 (13.2)	6 (15.8)
*Klebsiella pneumoniae* (41)	27 (65.9)	11 (26.8)	3 (7.3)
*Pseudomonas aeruginosa* (19)	13 (68.4)	3 (15.8)	3 (15.8)
*Escherichia coli* (5)	3 (60.0)	2 (40.0)	0 (0)
*Citrobacter freundii* (3)	1 (33.3)	0 (0)	2 (66.7)
*Providencia stuartii* (3)	1 (33.3)	0 (0)	2 (66.7)
*Serratia marcescens* (1)	0 (0)	1 (100.0)	0 (0)
*Elizabethkingia anophelis* (1)	0 (0)	0 (0)	1 (100.0)
*Stenotrophomonas maltophilia* (1)	0 (0)	0 (0)	1 (100.0)
Total (n= 112)	72 (64.3)	22 (19.6)	18 (16.1)

**Figure 3:**
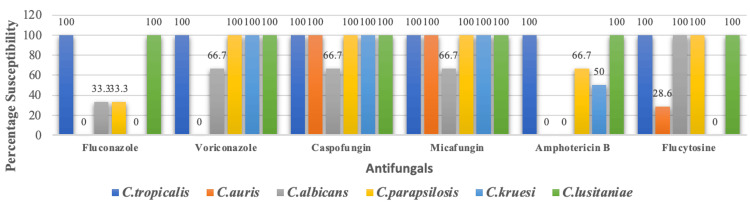
Antifungal sensitivity pattern of fungal organisms

Among the fungal isolates, C. lusitaniae and C. tropicalis displayed 100% susceptibility to all antifungal drugs tested. Candida auris exhibited complete sensitivity to micafungin and caspofungin. But neither C. albicans nor C. auris were sensitive to amphotericin B. For C. auris and C. kruesi, fluconazole exhibited 100% resistance (Figure 3).

## Discussion

Bloodstream infection pathogens can be fatal; they must be identified and subjected to antimicrobial testing as soon as possible [11]. It has been observed that delaying therapy initiation by an hour can result in an average decrease in survival of 8% [12]. In addition, when patients in the ICU with septicemia are administered inadequate empirical treatment, their mortality rates double from 30% to 60% [13]. The current study had a 31.8% culture-positive rate. Several Indian studies support this rate of isolation [3,14]. Some studies show a lower culture positivity [11,15]. However, a greater frequency of blood culture-confirmed cases (50.58%) was noted in another investigation [16]. Variations in culture positivity rates could be due to differences in blood culture methods (automated or manual), geographical location, the number of blood culture bottles taken, and the volume of blood used [17]. In our study, 52.6% of gram-negative organisms and 35.2% of gram-positive organisms were found. A different Indian study found that 51% of the isolates were gram-negative [18]. However, other studies have shown a higher prevalence of gram-positive organisms [19]. This disparity might have been influenced by the variations in epidemiology and the range of underlying causes.

The increased incidence of gram-negative bacteria may be related to hospitalized patients acquiring an infection. Their capacity to remain on surfaces for extended periods makes it easier for them to spread throughout the hospital environment. Furthermore, the hands of medical professionals are a major source of these bacteria, which cause outbreaks frequently. Our study's major bacteria were CoNS, accounting for 21.6%, which is comparable to the findings of Rao et al. [20]. For the past 20 years, CoNS has been frequently identified as a significant bloodstream pathogen [21]. It has been determined that inappropriate blood collection practices and prolonged use of intravascular devices are potential pathways for CoNS to spread BSIs [22].

In our study, candidemia has been confirmed in 12.2% of cases. The outcome is consistent with research done by Pal et al. [23]. In additional research, 7.9% had candidemia [24]. Non-*Candida albicans *species were responsible for the majority of candidemia infections in the study, consistent with previous research [25]. Our finding differed from that of Siddiqui et al., who found *C. albicans* to cause most BSIs [26]. The current research shows that MRSA accounted for 77.6% and methicillin-resistant CoNS consisted of 66%. Vanitha et al. suggest that the increased prevalence of MRSA and methicillin-resistant CoNS strains identified today could be attributed to the frequent overuse of third-generation cephalosporin as an empirical therapy in hospitals [21]. The results of our investigation, which are consistent with those of Nyesiga et al., show that gram-positive organisms have increased resistance to erythromycin, ciprofloxacin, and gentamycin [27]. Additionally, as reported by other authors, the study demonstrated that vancomycin and linezolid were both 100% effective against the CoNS and *S. aureus* [21]. In line with the findings of Birru et al., 84.1% of *Enterococcus* species were found to be sensitive to vancomycin [28]. In our study, 77.8% of isolates of *Enterococcus* were high-level gentamycin-resistant, which is higher than the study done by Palewar et al. [11].

This study, like previous ones, reveals that gram-negative bacteria are pandrug resistant than gram-positive bacteria [29]. This might result from using empirical medicine inappropriately as a first line of treatment. For clinicians, this is concerning since it leaves them with very few options for drugs, such as tigecycline and colistin, which have hazardous side effects [29]. In our study, the percentage of ESBL producers was 19.6%, which is lower than the 39.6% observed by Gohel et al. [30]. The percentage of MBL producers was found to be 64.3%, higher than the 60% reported by Shah et al. [19].

Limitations

It is unclear whether the infections in the participants were acquired within the community or the healthcare setting because of a lack of information. As this is a single-hospital-based retrospective analysis, it's possible that the findings cannot be applied to a larger population.

## Conclusions

This study found a considerable degree of antibiotic resistance, particularly in gram-negative bacteria. Inadequate infection control practices and excessive antibiotic use may be responsible for the rising incidence of antimicrobial resistance in the area. Thus, to control and prevent drug resistance successfully, extensive surveillance, the formulation of antibiotic policies, and preventive actions are required.
